# Object Segmentation from Motion Discontinuities and Temporal Occlusions–A Biologically Inspired Model

**DOI:** 10.1371/journal.pone.0003807

**Published:** 2008-11-27

**Authors:** Cornelia Beck, Thilo Ognibeni, Heiko Neumann

**Affiliations:** Institute for Neural Information Processing, University of Ulm, Ulm, Germany; University of Southern California, United States of America

## Abstract

**Background:**

Optic flow is an important cue for object detection. Humans are able to perceive objects in a scene using only kinetic boundaries, and can perform the task even when other shape cues are not provided. These kinetic boundaries are characterized by the presence of motion discontinuities in a local neighbourhood. In addition, temporal occlusions appear along the boundaries as the object in front covers the background and the objects that are spatially behind it.

**Methodology/Principal Findings:**

From a technical point of view, the detection of motion boundaries for segmentation based on optic flow is a difficult task. This is due to the problem that flow detected along such boundaries is generally not reliable. We propose a model derived from mechanisms found in visual areas V1, MT, and MSTl of human and primate cortex that achieves robust detection along motion boundaries. It includes two separate mechanisms for both the detection of motion discontinuities and of occlusion regions based on how neurons respond to spatial and temporal contrast, respectively. The mechanisms are embedded in a biologically inspired architecture that integrates information of different model components of the visual processing due to feedback connections. In particular, mutual interactions between the detection of motion discontinuities and temporal occlusions allow a considerable improvement of the kinetic boundary detection.

**Conclusions/Significance:**

A new model is proposed that uses optic flow cues to detect motion discontinuities and object occlusion. We suggest that by combining these results for motion discontinuities and object occlusion, object segmentation within the model can be improved. This idea could also be applied in other models for object segmentation. In addition, we discuss how this model is related to neurophysiological findings. The model was successfully tested both with artificial and real sequences including self and object motion.

## Introduction

Humans can easily segment objects that are moving in a scene. Whether a pedestrian is walking on a crowded sidewalk, or a driver wants to pass another vehicle, other moving objects can be detected without any effort. However, from a technical point of view the segmentation of moving objects is difficult to handle. Without knowledge of the background positions, the background motion cannot be computed, while without knowing the background flow we cannot determine which positions belong to the background region. For this reason, in the literature this issue is often referred to as a chicken-and-egg-problem. There are several approaches for how to deal with the problem of scene segmentation based on motion, such as the global parametric motion models [1]–[3]. Other models tend to find regions containing locally smooth motion that are surrounded by motion discontinuities [4]–[6].

**Figure 1:**
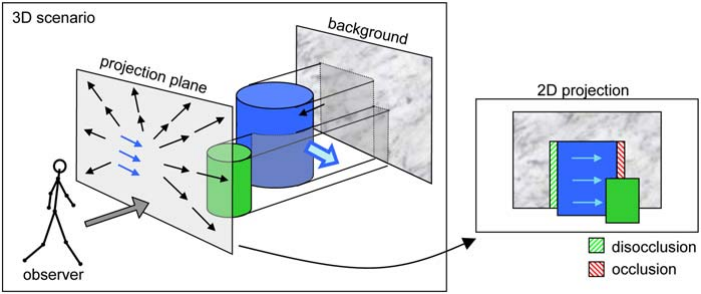
3D scenario with two objects. This figure depicts a typical scenario for a person moving in a room. A static object (green) and a moving object (blue) are located in the room in front of the background. On the left, static occlusion regions with respect to the observer perspective are marked with gray overlay. Due to the spatial configuration the green object is partly covering the blue one, both objects are occluding the background texture. When the observer is moving forward, an expansional flow field is generated that is partly superimposed by the translational movement of the blue object. The optic flow, i.e. the projection of the 3D flow is shown on the projection plane. The alignment of the objects in the 2D projection is shown on the right. Here, also the kinetic occlusions generated by the movement of the blue object are depicted. On its left side, background texture is uncovered (disocclusion), on the right side it is temporarily covered (occlusion). Note, that the expansional flow leads to further kinetic occlusion regions along the outline of both objects, for simplicity this is not included in the sketch.

Many models use the principle of “optic flow”, this being the 2D projection of the flow vectors onto the image plane relative to the observer, instead of the detection of the 3D motion in space (see Fig. 1). Different techniques exist to detect the flow vectors. Common approaches are the use of spatio-temporal derivatives or correlation-based algorithms that try to find similar patterns of the image in subsequent frames. Flow is basically generated by two different kinds of motion. First, self motion is due to movement of the observer, which results in global flow fields. Second, parts of the visual field can move independently leading to a locally different flow. These regions are referred to as independently moving objects. For a segmentation of the scene based on optic flow, the parts of the image moving in different ways have to be identified and grouped together. Motion boundaries (“kinetic boundaries”) that are at locations where different motion cues meet, are an important source of information to achieve segmentation. Unfortunately, the detection of optic flow is complicated at these positions as spatial integration of local flow may mix the different motion cues and thus lead to erroneous detection. Even for correct optic flow detection, segmentation simply based on the similarity of the optic flow will not be successful for all scenes. Depending on the kind of motion pattern in the sequence, regions of coherent optic flow contain different optic flow vectors, e.g., for an expansional movement. This can be solved by detecting the changes in the local flow field, called the motion discontinuities, rather than the smooth regions.

Occlusions play a particular role in the task of detecting motion boundaries. They appear when an object in front is moving in a different way than its surround. This can either be caused by a static background and a moving object, by a moving background and a static object, or by movement of both background and object. As a consequence, parts of the background–either other objects or background texture–are temporally covered by this particular object (“occlusion regions”). When the object moves further on, these regions are disoccluded again, but other regions will be covered. At first sight, occlusions only seem to complicate the detection of motion boundaries in an optic flow based approach. In occlusion regions no local matches for motion detection can be found and the intensity is not constant in space-time. This facilitates the effect of “motion bleeding”, when salient motion of adjacent regions is propagated into regions with few reliable detections. However, the explicit detection of occlusion regions generated by moving objects (“kinetic occlusions”) can also support the segmentation as occlusion regions are a clear hint for an object moving in a different way than its background. The detection of these regions can be achieved by looking for positions where no optic flow has been found [2] or by evaluation of the spatio-temporal structure [6]. Detecting occlusion and disocclusion regions is also interesting for further interpretation of the scene, as it allows the assignment of a relative local depth order [2], [4], [7].

The analysis of optic flow can be described as an *estimation* problem. Such an estimation process is defined by different components and the results are influenced by different parameters. At first, the detection of motion consists of a decision about whether movement is present at a location or not. Second, the measurement of specific attributes of the motion is defined by the velocity, which is composed of speed and direction. Finally, a confidence value of the measurement defines the reliability of the measurement, or estimation, process. In our approach presented here, the activities of model neurons reflect a confidence value or a likelihood for the velocity for which they are tuned. This is due to the correlation-based approach used here, as the process of *detecting* the optic flow is invariant to luminance contrast. In other words, the activity of a neuron representing a particular motion only depends on the movement itself and is not confound by possibly varying local luminance under changing scene illumination.

We propose a biologically inspired model for object segmentation that includes processing components for motion detection, and in contrast with previous approaches, makes use of both motion discontinuity and occlusion detection. The motion detection itself can handle the problems that complicate flow detection at occlusions due to the representation of more than one motion locally and a mechanism to get reliable motion detection also in occlusion regions. The computation of motion discontinuities and occlusions is effected in different components using two different mechanisms, based on spatial and temporal contrast detection, respectively. The crucial functionality within this model consists of the feedback connections between its components which enable the transfer of information. Our results show that the segmentation of moving objects can be considerably improved if occlusion and motion discontinuity detection mutually interact. Temporal integration of information is applied in these model components to make the results more stable. Furthermore, the model results for occlusion and disocclusion regions, as well as the segmentation that was achieved, is further processed for an interpretation of the scene. Both ordinal depth order (spatial order of objects in a scene) and the local differences between object and background movement are computed. This represents an important step towards the goal of reliable segmentation of independently moving objects in a scene.

## Methods

### Motion processing in the brain

From extensive research of the visual processing in the human brain it is known that the spatio-temporal stimuli impinging the retina are processed subcortically, and are then projected to the primary visual cortex. From here two major pathways realize the further processing that is thought to compute specific stimulus properties [8]. Early and mid-level motion analysis in visual cortex is primarily associated with the dorsal pathway that generates the main input to the “Where system” [9], including the primary visual cortex (V1), the medial temporal (MT), and the medial superior temporal area (MST). Form information is mainly processed in the ventral pathway that generates the main input to the “What system”, including areas V1, and visual areas V2, and V4. There is also an exchange of information between the two pathways via many connections between different areas.

Motion processing starts at the early stage of primary visual area V1. Stimuli there are analyzed in parallel for movement direction [10]. Primary visual cortex projects to MT in a feedforward fashion and receives feedback connections from MT. In MT, neurons exist to build a more detailed representation of two-dimensional image velocity, namely direction and speed [11]. The output of the optic flow computation in MT provides input to the MST subdivision of the motion sensitive complex, MSTl and MSTd, respectively. Area MSTl is primarily concerned with object motion, i.e. the detection of spatial motion contrast through center-surround processing of motion fields, with different directions and their spatial segregation being based on disparity information [12].

Concerning the form processing, neurons have been found in V1 that respond to oriented contrast. The input is passed to area V2 where long-range filters perform a grouping of elongated contours [13].

### Overview of model components

In our model we make use of different processing stages that are mainly inspired by the findings summarized in the previous subsection. Most of them can be grossly associated with mechanisms found in these cortical areas, for this reason they are named after the corresponding area. At the current state, some of the mechanisms included in the model can not be attributed to particular areas, therefore they are denoted according to their functionality.

Preprocessing in V1_Model_ is accomplished by detecting initial motion as well as local contrast. The detected motion information is fed forward to MT_Model_, MSTl_Model_, and the component for the detection of temporal occlusion, TO_Model_. In these three components, motion integration, motion contrast, and occlusion detection is accomplished in a network of mutually interacting sub-populations of model neurons.

**Figure 2:**
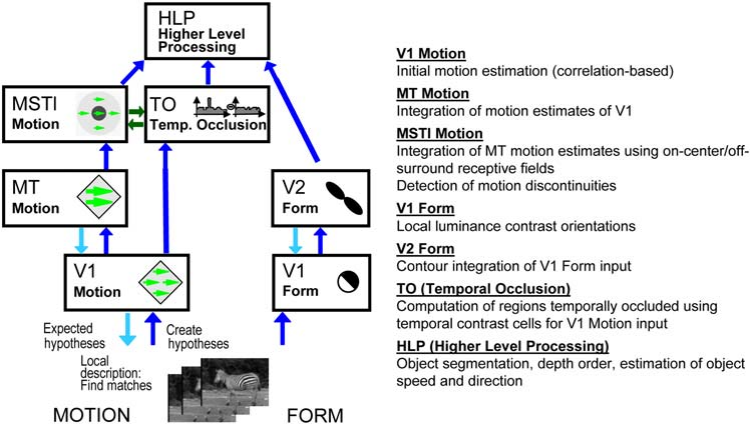
Sketch of the biologically inspired model. V1_Model_ Motion and MT_Model_ Motion represent the basic modules for optic flow estimation. In TO_Model_ regions that have been occluded or disoccluded are estimated. In MSTl_Model_ motion discontinuities are computed based on MT_Model_ input due to spatial on-center-off-surround receptive fields. The information of areas MSTl_Model_, TO_Model_, and V2_Model_ is combined in a higher level processing area (HLP_Model_). Feedforward connections are depicted with dark blue arrows, feedback connections with light blue arrows. The interactions between MSTl_Model_ and TO_Model_ are depicted with green arrows.

The form information detected in V1 is fed forward to V2_Model_, where extended boundaries are extracted by mechanisms of long-range integration. In addition, the model includes a higher-level processing component (HLP_Model_) that integrates the output generated at the lower stages of processing. In HLP_Model_ information generated by MT_Model_ and MSTl_Model_, as well as available boundary information represented in V2_Model_, are integrated to obtain a segmentation based on optical flow and relative depth order of scenic objects. Note that HLP_Model_ and TO_Model_ are not linked to a specific cortical area. Figure 2 shows an overview of the model components and their connections. In the following subsections, the different parts of the model will be introduced in more detail.

### Feature detection and integration: Motion analysis in V1_Model_/MT_Model_ and form processing in V2_Model_

In our model, the interplay of V1_Model_ and MT_Model_ is one crucial aspect to achieve robust detection of optic flow, e.g., to solve the aperture problem. Our model parts for optic flow detection, V1_Model_ Motion and MT_Model_ Motion, are based on the approach of Bayerl & Neumann [14]. They developed a fast algorithmic version of their previously proposed neural model of motion perception [15], in which a sparse representation of stimulus motion (local velocities) is used and further refined.

#### Initial motion detection

The input stage to V1_Model_ consists of an initial motion detection that is a correspondence based approach measuring the frame-to-frame similiarity of the local image structure. Such a description can be achieved using a variation of the Census Transform [16] or a combination of different derivative filter responses. In both cases, at each position a bit string (“feature value”) is computed that describes the local image structure. To detect the motion for two frames t_1_ and t_2_, the feature values are computed for each position. If two positions p_1_ in t_1_ and p_2_ in t_2_ have the same feature value, movement from p_1_ to p_2_ can be assumed, because the local image structure is the same. The search for these correspondences can be realized algorithmically in an efficient way using sorted tables of the feature values of both input frames. The motion vectors that are found during this process are then saved as “hypotheses”, i.e., a structure consisting of a position, a velocity, and a weight. To achieve a sparse representation, hypotheses are only created for feature values that appear at only few image positions (we use h_max_ = 5). If the same feature value can be found at many image positions, the motion estimate is very ambiguous as many corresponding matches can be found, leading to a huge number of hypotheses that are hardly reliable. Therefore, we only use the feature values that are salient because they appear at few positions. One exception for this procedure is in the case of feedback. If feedback from MT_Model_ predicts a certain movement, we will generate a new hypothesis even if the feature value can be found often, with an upper limit of H_MAX_ (h_max_≪H_MAX_). The hypotheses generated in this first step are then used as input to the processing hierarchy.

#### Optic flow detection

In the processing hierarchy of the model, V1_Model_ is representing raw and rather noisy estimates of the optic flow with a very high spatial resolution that are integrated in MT_Model_ leading to more reliable estimates, but reduced spatial accuracy. The integrative fashion of the forward processing path is indicated by increasingly larger receptive field size (neurons that provide input), with a ratio of approximately 1∶5 for V1_Model_∶MT_Model_ [17]. Both components communicate using a bidirectional flow of information, i.e. the feedforward stream is augmented by a reverse signal flow via feedback. Such feedback is mainly modulatory in its effect such that existing input activity is enhanced while feedback alone cannot generate new activity [18], [19]. In our simulation, feedback connections are incorporated using the “linking principle” proposed by Eckhorn et al. [20].

The simulation of neural processing within the components follows a general principle of a three-level-processing cascade that has been successfully applied for other models in visual processing, e.g., texture boundary processing [21] and contour integration [22]. In particular, each of the model components is defined by linear and non-linear computational stages:

1. Feedforward integration via linear or non-linear filtering of input feature activations. This processing acts as a driver feeding the system with sensory signals.
2. Feedback to neurons in an earlier component is modulatory such that neural activations from higher model components amplify activities in an earlier component (gain control). The enhancement of activities by more global context information leads to a bias giving the corresponding features a competitive advantage in the subsequent center-surround processing.
3. Lateral shunting inhibition based on divisive on-center-off-surround competition to normalize activities in a pool of neurons and to enhance salient signals. The mutual interplay between excitatory feedback and mutual inhibition leads to increased responsiveness to target object detection and a decrease in background response [23].

The dynamics of the individual stages was defined formally by using first-order ordinary differential equations, utilizing single-compartment neuron models at the individual processing stages. In particular, we have
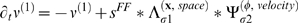
(1)

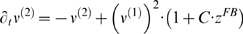
(2)

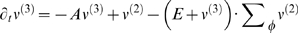
(3)Eq. 1 describes the initial filtering stage to generate the input of the particular model component. In Eq. 2 the linking mechanism of the modulatory feedback is implemented. The activation of the previous stage serves as input that is transformed by a non-linear signal function (we use squaring non-linearity). The activity z^FB^ denotes the feedback signal from higher level stages of the processing hierarchy that is amplified by a constant C. The term (*ν* ^(1)2^·(1+C·z^FB^) ensures that the input activation (driving signal) is enhanced by the feedback signal. If no feedback signal is provided the driving input is passed forward unchanged. However, if no feedforward signal is generated, feedback alone cannot generate any new activity. The final stage is denoted by Eq. 3 implementing an on-center-off-surround mechanism in velocity space. Here, an individual activity in space-feature domain, e.g., velocity, competes against the sum of activations for all velocities at the particular location. The term (E+*ν* ^(3)^) denotes a multiplicative term that shunts the inhibitory input. The effect can be identified by the steady-state solution of Eq. 3, namely *ν* ^(3)^_inf_ = (*ν* ^(2)^−E Σ*_φ_ν* ^(2)^)/(A+Σ*_φ_ν* ^(2)^).We observe that the constant E weights the component of linear subtractive inhibition in the numerator, while the self-inhibition by *ν* ^(3)^ leads to a net divisive effect (denominator). The constant A is the rate of decay of the activity.

#### Boundary processing

In addition to components for motion processing, we also simulated components to include form information in the model. This information can be used to achieve object boundaries defined by a strong luminance contrast at high spatial resolution and thus to complement motion boundaries extracted in MT_Model_/MSTl_Model_ as explained in the next subsection. Also, form information is helpful for the grouping of motion boundaries. When two objects overlap, they typically form a “T-junction”. These T-junctions can be detected using form information. Grouping should then be restricted at these positions to avoid two objects being integrated into one.

The form information is computed by our model in two recurrently connected components V1_Model_ Form and V2_Model_ Form. In V1_Model_ Form, the local luminance contrast is computed for eight different orientations, in V2_Model_ Form, V1_Model_ responses are used as input to bipole filters composed by two anisotropic Gaussian filters that are combined in an additive way. This kind of filters extracts salient elongated contours of the input image. Object contours can be found using the two model components. In both components the same processing cascade as presented in the previous subsection is applied. To achieve a robust estimation of the contour, some iterations including feedforward and feedback connections between V1_Model_ and V2_Model_ are necessary. A measure of local junctions is computed by evaluating the presence of orientation responses at each spatial location. High responses for orientations arranged like a “T” indicate the presence of an object occluding another.

### Detection of motion boundaries in MSTl_Model_

#### Detection of motion discontinuities

**Figure 3:**
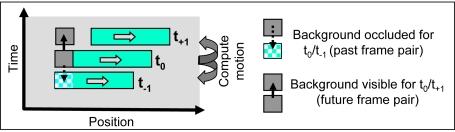
Optic flow estimation at occlusions. Occlusions lead to problems for motion estimation algorithms based on the correlation between only two frames: Parts of the image are only visible in one of the frames, thus no corresponding image positions can be found at these locations. This problem can be solved using only one additional temporally forward-looking step (future step).

In our model, MSTl_Model_ is primarily concerned with object motion, i.e. the detection of spatial motion contrast through center-surround processing of motion fields with different directions (Fig. 3). These neurons receive input from MT_Model_. They are highly activated if the movement presented in the central part is different from the movement in the surround and are thus tuned to motion discontinuities, i.e. positions where two or more movements meet.

For the integration of this mechanism in the architecture, we modelled MSTl_Model_ neurons that obey an on-center-off-surround characteristic generated by input integration from model MT_Model_ neurons. To reduce the computational complexity the mean velocity estimated at each position is used by taking the sum over all velocities (v_x_, v_y_) at one MT_Model_ location where each discrete measure is weighted by its respective activity *u* **_x_** ^MT^. In computational terms the mean flow vector *v̅*_x_ at position ***x*** is determined by
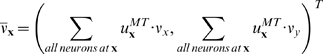
(4)In MSTl_Model_, the on-center receives input from one neuron, whereas the off-surround comprises a larger spatial neighbourhood (5×5 positions in our simulations). If the mean velocity at a surround position is similar to the mean velocity in the center, this will contribute to the inhibition of the overall activity of the neuron. For this purpose, the activity at the surround position is weighted with a spatial Gaussian function. Spatial contrast responses *w* **_x_**_Δv_^MSTl^ are computed by the following equation
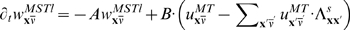
(5)In the simulations we set A = 1, B = 1, Λ^s^ is a Gaussian kernel to weight the activity in the spatial surround. Temporal integration can be used to stabilize the results of MSTl_Model_. For this purpose, the motion discontinuities of the last time steps are shifted to the current position (based on the object velocity of the object they belong to), and then added to the current motion discontinuity value. The influence of current and past frames is determined by a weight function that decreases with temporal distance. A moving average is used for an efficient computation of the temporal integration:
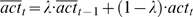
(6)After the computation of the motion discontinuities, further steps are necessary to obtain an explicit segmentation of the scene. As these mechanisms are currently not in the focus of our biologically inspired approach, we use a simple grouping and filling-in mechanism to derive a segmentation of the scene based on the motion discontinuities. Employing the results of the segmentation, a mean velocity for all detected objects can be computed by summing up the mean velocities for all positions belonging to an object. As we do not assume a simple translational movement over all the background, a global motion estimation derived by summing up the single flow components of the background positions would not provide a reasonable approximation.

#### Occlusion detection

**Figure 4:**
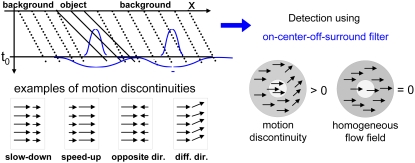
Detection of motion discontinuities. Some examples for motion discontinuities are given on the left bottom. We use a motion discontinuity detector built of an on-center-off-surround RF that will respond very strongly if center and surround motion differ. If a homogeneous flow field is presented, only a weak response is produced.

**Figure 5:**
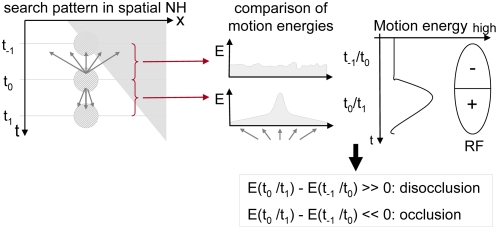
Detection of occlusion regions. To detect occlusions and disocclusions in the motion sequence, we compare the motion energy at each spatial position that was estimated using the past frame pair t_−1_/t_0_ and using the future frame pair t_0_/t_1_. A high difference typically occurs at occlusion and disocclusion positions due to regions that are only visible in t_−1_ or t_1_ and thus entail very ambiguous motion estimates.

The generation of reliable motion detection at motion boundaries is a difficult task, for in the occlusion regions the detection of corresponding local image structure is not possible for frame t_−1_ and t_0_. The lack of local estimates has the consequence that in these regions motion bleeding can appear. This means that salient estimates of the neighbourhood, like of the object generating the occlusion, propagate into the occlusion regions. The propagation can be limited if the motion estimates within the occluded region are strong. For this purpose, we extended the model for motion detection by a mechanism of temporal integration [24]. The underlying idea is that motion estimates within t_−1_/t_0_ (“past frame pair”) will fail to calculate the correct optic flow for the image regions containing occlusions. The past frame t_−1_ contains occlusion regions where parts of the background are covered, while they are visible in frame t_0_ (see Fig. 4). This problem can be solved by using motion cues of one additional future frame to compute the correspondences between t_0_ and t_1_ (“future frame pair”), where the occlusion regions are visible in both frames (assuming coherent motion for the object). The estimates of the two frame pairs are then used as parallel input to V1_Model_. The occlusion regions are so mainly filled with estimates from the future frame pair as the past frame pair will not contribute a large number of motion estimates at these positions. For the disocclusion regions, mainly the input from the past frame pair is important. Using this specific property of occlusions we are able to compute reliable estimates for occlusion regions without using an explicit detection of these regions. This mechanism offers therewith a good basis for ongoing higher evaluation relying on dense and stable optic flow, like in MSTl_Model_. On the other hand, the activity provided from the different frame pairs can now be further processed by appending neurons for the detection of occlusion and disocclusion regions. The model is extended by a temporal on-center-off-surround mechanism that responds strongly if at the local position a change in motion energy appears. A change of local motion energy is a strong cue for occlusions as the non-matchable points in an occlusion region entrain low motion energy locally. Temporal motion contrast neurons that respond strongly for changes from low motion energy to high motion energy indicate disocclusion regions, temporal motion contrast neurons that detect changes from high to low motion energy indicate occlusions (Fig. 5). The motion energy at each position is computed by summing up the number of hypotheses generated in a small spatial surround. The following equation describes how the activity in TO_Model_ is computed at time t_0_:
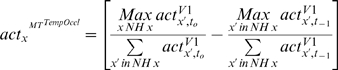
(7)This processing step is accomplished after feedback from MT_Model_ supported the creation of motion hypotheses. The computation is very cheap as the main extra effort is the computation of the difference of motion energies (see Eq. 7).

Note, that due to the way the occlusion and disocclusion regions are computed, these regions will both appear spatially outside the occluding object, i.e. in the background. Using this detector at each image position, we can assign an occlusion activity for each position. To allow further analysis of the image, like finding the object that caused the occlusion region, we employed a simple grouping mechanism to get a common label for each occlusion and disocclusion region. For this purpose, adjacent occlusion and disocclusion positions (occlusion activity bigger than a threshold) were pooled to groups of occlusions and disocclusions, respectively, and then provided with a label.

To stabilize the results of the occlusion detection, we use a temporal integration for the occlusion regions. When the integration is computed, the change of spatial position during time has to be considered, leading to a spatial shift of the occlusion regions computed in the last time step by the motion of the corresponding object. Like for the MSTl_Model_ neurons detecting motion discontinuities, a moving average is used to compute the temporal integration in an efficient way.

#### Interactions of occlusions and motion discontinuities

In the previous subsections, mechanisms to reliably detect motion discontinuities and occlusion regions were presented. Both motion discontinuities and occlusions are computed using on-center-off-surround neurons. The detection of motion discontinuities is represented by local motion changes, whereas the detection of occlusion is based on temporal changes of motion energy. Nevertheless, there is an important connection between the two features in the context of object detection: motion discontinuities usually entail occlusion regions. In other words, a motion discontinuity is generated by an object that moves in a different way than its neighbourhood. For this reason, it inherently produces occlusions. This means, that we can use the detection of motion discontinuities to support the position where occlusion regions are found and vice versa. We included this link in the model via mutual excitatory multiplicative feedback connections between MSTl_Model_ and TO_Model_. The feedback plays an inhibitory role. Motion discontinuities that are not overlapping partly with occlusion regions are eliminated as they are probably an erroneous estimate. If this mechanism is used, factor *B* in Eq. 5 will depend on the activity of TO_Model_ neurons. Responses in TO_Model_ are modulated by MSTl_Model_ feedback, activity at positions that do not get support from MSTl_Model_ is strongly reduced (right side of Eq. 7 multiplied with a factor C = 0.01+FB^MSTl^).

### Higher-level processing

**Figure 6:**
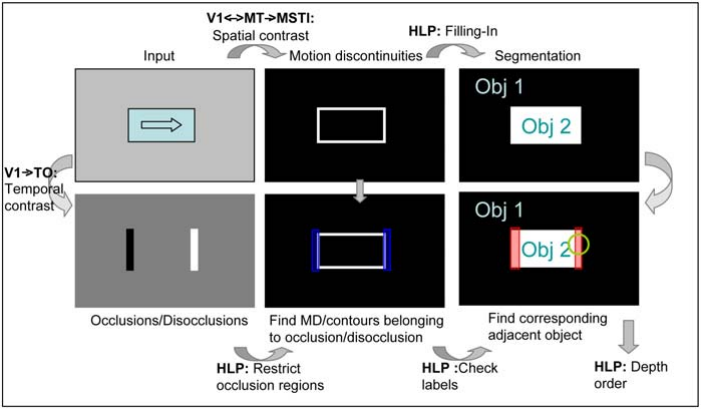
Overview of mechanisms for scene interpretation. Top row: The optic flow of the input image is computed in V1_Model_ and MT_Model_, spatial contrast neurons in MSTl_Model_ compute the motion discontinuities. Based on the detected motion boundaries a simple filling-in mechanism provides a scene segmentation. Bottom row: In TO_Model_ input from V1_Model_ neurons is used for a temporal on-center-off-surround processing step to detect occlusion and disocclusion regions. In HLP_Model_ these regions are restricted to the motion discontinuities or luminance contours provided from V2_Model_ to find the corresponding object that is adjacent to the occlusion region, namely the occluder. The results of the object segmentation are used to find the label of the corresponding object (indicated by the arrow from the top row, third column). Based on these data, the corresponding depth order can be computed. Interactions between MSTl_Model_ and TO_Model_ are not depicted in this figure.

To achieve an interpretation of the scene, the information of the different processing stages has to be combined in an integrative way. We aim at the segmentation of the images based on the information from V1_Model_/MT_Model_ and MSTl_Model_ and the derivation of an ordinal depth order. For this purpose, depending on the largest overlap of each occlusion region and the object at this position, the occlusion regions can be related to their corresponding object. Then, the object that caused the occlusion can be identified by checking the object labels along the motion discontinuities that are close to the occlusion region (see Fig. 6).

For objects where a clear object outline can be detected due to salient local luminance contrast in the form channel using V1_Model_ and V2_Model_ Form, the motion boundaries can be sharpened. As these contours are computed at high spatial resolution, they help to find the exact local position of the boundary for an object detected by MSTl_Model_ center-surround neurons for motion discontinuity detection. If no contours can be found, e.g., for a movement of dot patterns, we can simply rely on the motion discontinuities leading to a coarser localization as the spatial resolution of MSTl_Model_ is less accurate than in V1_Model_/V2_Model_ due to larger integration steps in the feedforward processing from V1_Model_ to MT_Model_ and MT_Model_ to MSTl_Model_, respectively.

## Results

In this section we present the results of our model for both artificial and real image sequences. In the focus of our work is the detection of motion discontinuities and occlusions for reliable optic flow segmentation that is further improved by interaction between the two features. To demonstrate that the approach is working independently of the scenario we will show results of experiments with different kinds of global and local movement. Based on the segmentation and the detected occlusions, the ordinal depth order in the sequence is determined. The size of the input images used was approximately 320 by 240 pixels (depending on the scene), the mean computing time for one iteration was between 3.5 and 5 seconds using a standard CPU (Athlon 2000 GHz, 1 GB RAM). The current implementation (C++) is not optimized for real-time processing. We claim that the same results can be achieved in real-time/close to real-time, if GPU routines and speed optimized algorithms are used. The results shown in this section were computed using one “in place” processing step with the same input frames as before (i.e., t_−1_, t_0_, t_1_) to further stabilize the results, followed by an iteration including a new frame (i.e., t_0_, t_1,_ t_2_).

In the following subsection, we will a) show the results for motion discontinuity and occlusion detection, b) provide examples for object segmentation and estimation of the ordinal depth order, and c) demonstrate the effects of interactions between MSTl_Model_ and TO_Model_. If interactions between these two components were used, it is explicitly mentioned in the text or in figure captions.

### Detection of motion discontinuities and occlusion regions

**Figure 7:**
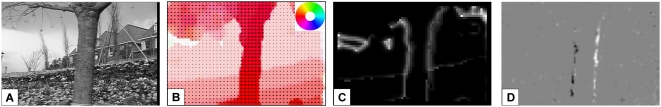
Experiment 1: Flowergarden sequence. A) Input image. B) Optic flow estimated in area MT_Model_, direction is indicated by a color code, speed by the corresponding saturation. C) Motion discontinuities appear due to the faster optic flow on the tree and along the regions where no movement is indicated as for the sky. D) TO_Model_ responds strongly along the contours of the tree trunk as during the translational self-motion the trunk occludes parts of the background (white color indicates disocclusion areas, black color occlusion areas). The results shown here include feedback from MSTl_Model_ neurons.

In the first experiment the flowergarden sequence (obtained from www.bcs.mit.edu/people/jyawang/demos/garden-layer/layer-demo.html, [25]) is used as input to the model (see Fig. 7A). The sequence shows a tree in front of houses and a garden passing to the left (at different distances) as the observer is making a translational movement to the right. The motion parallax leads to slower motions for objects further away from the observer. Therefore, the faster movement of the tree in front leads to occlusion regions in particular along the tree trunk. In Fig. 7C and D the detected motion discontinuities and occlusion/disocclusion regions are shown. Model neurons in TO_Model_ correctly indicate disocclusions on the right side of the tree and occlusions on the left side. In the treetop only few occlusions are found as the background there is basically homogeneous, this makes the detection very difficult. In contrast, motion discontinuities are detected all along the outline of the tree. There are some outliers on the left due to the transition from the white region of the sky (not motion estimates found) to the garden. Both motion discontinuities and occlusions were detected in a stable way during the whole sequence. The results show the successful occlusion and motion discontinuity detection of a real sequence with translational self motion and objects at different distances.

### Object segmentation and ordinal depth order

**Figure 8:**
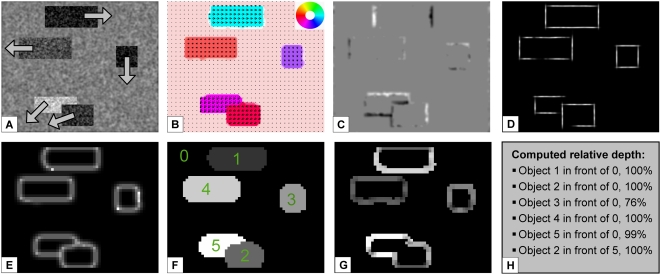
Experiment 2: Moving boxes. Results for an input sequence with 5 boxes and the background all moving in different directions. A) Input image with arrows indicating the movement of the objects. The background is slowly moving to the left. B) Mean optic flow estimations in area MT_Model_ marked with a color code that is superimposed on the input image. In C) the detected occlusion (black) and disocclusion (white) regions are shown. Note that depending on the direction of the object movement these regions appear all along the object boundaries or just on two sides (for a movement in vertical or horizontal direction). D) Contours of the objects as provided by V2_Model_ Form. This activity is used to achieve a clear localization of the occlusion boundary to the corresponding occluder. E) A clear segmentation of the object boundaries is achieved using the motion discontinuities detected with MSTl_Model_ on-center-off-surround neurons. F) After the detected boundaries have been grouped and filled, the image is segmented in different regions representing the objects of the scene. G) Classification of object movement. The difference of object and background motion is computed as explained in the Methods section. Light object boundaries indicate a strong difference, darker outlines represent a movement similar to the background. Note, that object 5 and 2 have a strong motion contrast to the background despite the similar movement direction due to a much higher speed than the background. H) The results of the relative depth order derived automatically from the scene. A confidence value is applied to get a probability for the correctness of the depth order (indicated in percent). This is derived from the number of positions belonging to the object that indicate that the object is in front (#pos_front_) and the number of positions that indicate that the object is in the background (#pos_bg_) (conf = max(#pos_front_, #pos_bg_)/(#pos_front_+#pos_bg_).

In a second experiment we investigated the question whether the model is able to segment objects moving in front of an independently moving background and whether ordinal depth order can be assigned correctly. We created an artificial sequence with several rectangles moving in different directions while the background is moving as well. To make the scene more complex, one of the objects is not only occluding the background, but also another object. The results for this sequence are shown in Fig. 8. All model components have accurate estimates, both motion discontinuities and occlusion regions are detected correctly. In Fig. 8F the segmentation based on the motion discontinuities is depicted. At the positions where one object is overlapping another, this is a more difficult task than for the other objects. The motion discontinuities of the two objects are mutually connected, a simple grouping approach would thus group the two objects together. To avoid this, we included information of the form channel. The grouping of the motion discontinuities is stopped at T-junctions as these indicate the junction of two objects. This means that the top of the “T” will not be grouped together with the stem of the “T”. In Fig. 8H the automatically derived ordinal depth order is indicated. For this artificial scenario the local object boundaries along the occlusion regions are all correctly estimated, also the occlusion regions are correctly assigned to the local background, even in the case of the two overlapping rectangles leading to the correct interpretation of relative depth order. A coarse classification of the object movement with respect to the background is depicted in 8G. For this task, we use the sum of the local motion contrast all along the detected boundary (square root of difference of optic flow). For an object moving with a similar velocity as the surround, this will result in a very small value (dark outline). If an object is moving in another direction than the background, the value will be much higher (light outline). For example, object 4 (compare numeration in 8F) has a similar movement compared to the background as indicated by the darker outline. Object 3 has a different direction, but a similar speed compared to the background, also resulting in a darker outline due to the measure of difference used (see Methods section). Our model can also detect the motion boundaries of objects that are simply defined by kinetic boundaries, i.e. objects that are not visible without movement. For example, the segmentation of the moving boxes as presented in Fig. 8 has basically the same results if the image texture is a random pattern in which the rectangles are moving. This is possible as the motion estimation itself can still find the local motion in V1_Model_ and MT_Model_, form information is only supplemental in MSTl_Model_ to find the motion boundary.

**Figure 9:**
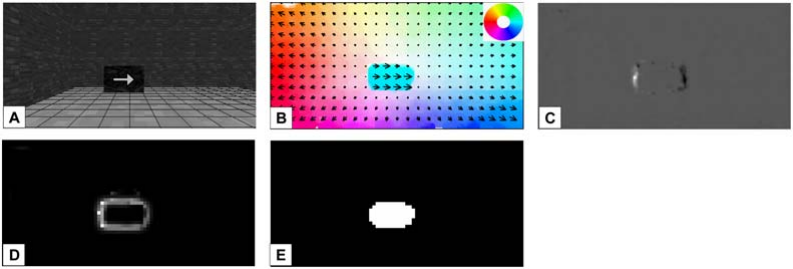
Experiment 3: Independently moving object in a scene with a moving observer. A) Input image of the sequence (generated in the XVR environment, download at www.vrmedia.it), the gray arrow indicates the movement of the independently moving object. B) The optic flow in area MT_Model_ is depicted, the object movement is correctly indicating a translation to the right. C) Occlusions and disocclusions are correctly detected on the right and left side of the object, respectively. The result shown here include feedback from MSTl_Model_. D) Motion discontinuities as computed by MSTl_Model_ on-center-off-surround neurons show the object boundary, E) after the grouping and filling-in step the object can be segmented.

In experiment 3 we simulated an observer moving forward generating a global expansional flow field in which one object is moving independently. This allows us to test whether the same mechanisms work if not only planar motion is contained in the scene. Based on the motion discontinuities, a first segmentation of the image is achieved. In contrast to approaches relying on segmentation via a similarity measure based on the optic flow itself, we can handle continuous changes of optic flow within an object without problems. This is important to correctly segment moving objects in 3D scenes while a strong expansional component occurs due to forward or backward movement of the observer. Figure 9 shows the estimated occlusion regions, motion discontinuities, and the object segmentation. Both occlusion mechanisms and MSTl_Model_ neurons correctly detect the corresponding regions, also in this scenario the moving object can be segmented and the ordinal depth order correctly indicates that the box is in front of the background region (not shown).

**Figure 10:**
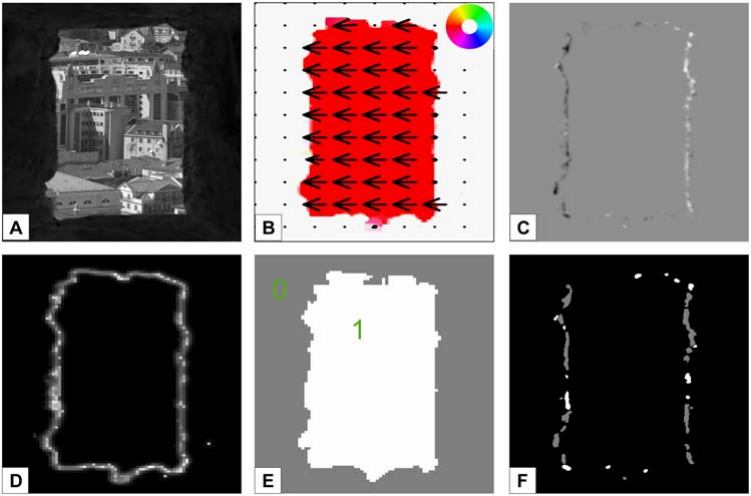
Experiment 4: City view through a window. Artificially generated scene with a background moving to the left while the aperture is fixed. A) One image of the input sequence. B) The mean optic flow as detected in MT_Model_. C) The movement generates occlusions on the left (black positions) and disocclusions on the right side (white positions). D) The motion discontinuities show the complete object boundary. E) After segmentation two objects are detected depicted in different colors, the aperture (gray) and the region within the window (white). F) The corresponding occluder to the occlusion positions with respect to the objects segmented like shown in E), the colors indicate the assignment. Most positions correctly indicate the aperture as the object causing the occlusion.

In experiment 4 the sequence contains a background that is seen through an aperture. This means that the aperture is now the occluding object which inverts the ordinal depth order if compared to the former experiments. The results depicted in Fig. 10 show the motion discontinuities along the aperture as well as occlusions on the left and disocclusions on the right side. This reflects the effects produced by the movement of the background from right to left. For each detected occlusion region we automatically assigned the object that produced the occlusion or disocclusion, to find the corresponding occluder. The results are shown in Fig. 10F, most of the occlusion regions are correctly assigned to the aperture, there are few exceptions that indicate the background. From these results, the ordinal depth order can be derived indicating the correct inverse order (object 0 in front of object 1).

### Interaction of MSTl_Model_ and TO_Model_

**Figure 11:**
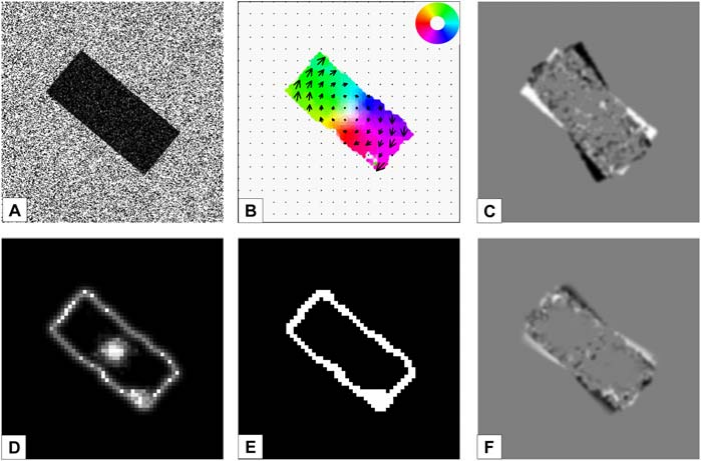
Experiment 5: Rotating rectangle. A bar is rotating around its center in front of a stationary background. A) Input image of the sequence. B) The motion estimates of area MT_Model_, C) Discclusion regions appear on the upper left and the lower right, in contrast occlusions are found at the lower left and the upper right, this diagonal appearance is due to the rotational movement of the object. The result indicated here is without feedback from motion discontinuities. D) The motion boundary is correctly detected using the motion discontinuities, however, also in the object center MSTl_Model_ neurons respond strongly when the movement switches from zero movement to the smallest movement that can be detected with the model. E) When including the interaction between occlusion and motion discontinuity detection, the erroneously detected central part is erased. F) Occlusion regions are correctly restricted due to feedback from motion discontinuity neurons as shown in D. The feedback is slightly blurred as occlusion regions may be significantly bigger than motion discontinuities.

In the subsection above we presented correct results for object segmentation based on motion discontinuities. However, for some input sequences motion discontinuities have the problem that they tend to oversegment the image, i.e. objects that do not exist are erroneously indicated. In particular for noisy input images, occlusions will not only be detected at the correct positions. In experiment 5 we investigated a sequence with a bar that is rotating around its center in front of a stationary background. Due to the fixed center point where zero motion is provided, the continuous transition to subpixel movement is hard to detect with optic flow algorithms like the one we use. This leads to an erroneous motion discontinuity around the central part as shown in Fig. 11D. When we now add a multiplicative factor from the detected occlusions as feedback to the MSTl_Model_ contrast neurons, this motion discontinuity can be eliminated. The erroneous motion discontinuity is in a region of the image where no continuous occlusions can be found, the interaction correctly deletes the generated segmented object. In Fig. 11E the object outline after interaction with occlusion neurons is shown. The effect of multiplicative feedback from motion discontinuities to occlusion regions is indicated in Fig. 11C and F. Without feedback many very small wrong occlusions are found in the image (11C), when the information is used as feedback, mainly the correct occlusion regions remain (11F).

**Figure 12:**
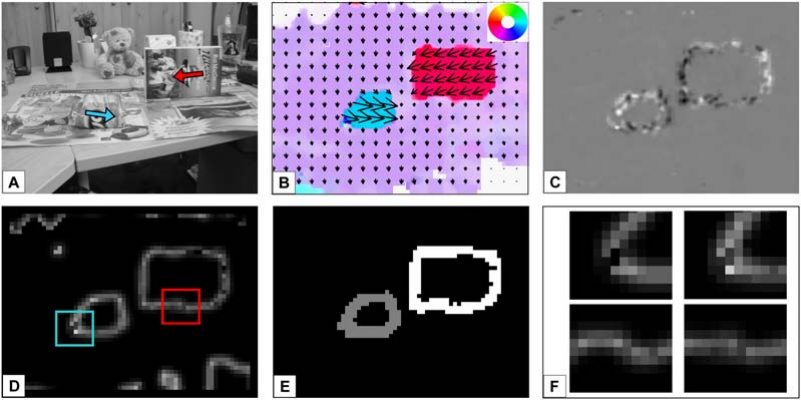
Experiment 6: Detection of moving objects in a real sequence. A) Input image of the sequence representing two objects moving in opposite directions and a translational camera movement upwards. B) Mean optic flow estimated in area MT_Model_, the direction of movement is depicted with the color code shown in the top right corner. C) In movement direction of the objects the dark region represents the occlusions detected, behind the objects white positions indicate the disoccluded region. Due to higher object speed the regions here are bigger than in the other experiments. According to the noise included in the scene, the estimates also get noisier, but still the overall response reflects the correct occlusion and disocclusion regions. D) The motion discontinuities including temporal integration (three frames used) clearly indicate the object boundary, E) after grouping the scene is segmented into background (black) and the two objects (gray and white). The motion discontinuities in D) in the upper left and the lower right part are not according to the results of the detected kinetic occlusion. The results in E) after the interaction with TO_Model_ thus correctly indicate only 2 objects. F) Comparison of motion discontinuity results without (left column) and with (right column) temporal integration. Without temporal integration the quality of the motion discontinuities is reduced: For expample, the gap in the smaller object at the lower left corner can only be closed using the temporal integration (first row, position indicated in light blue in D). Also the outline of the other object becomes straighter (second row, position indicated in red in D).

As the task of high quality optic flow estimation is more difficult in real image sequences than in generated scenes due to noise, shaking of the camera, etc., we used another real sequence in experiment 6 to test robust object segmentation. The camera in this scene is moving upwards, a book and a small box of cookies are moving from right to left and left to right, respectively. In Fig. 12 the results for this scenario are shown. Occlusion regions are correctly detected, the book generates occlusions at its left and the lower contour, the box generates occlusions in front and slightly along the lower contour. The results are noisier than in the scenes before, but still the correct detections prevail. The advantage of temporal integration for the motion discontinuity estimation is shown in Fig. 12F. Here, motion discontinuities with and without temporal integration are depicted for selected image regions (indicated by the colored boxes in 12D). To avoid long reaction times when the movement of one object is changing, the temporal integration should only include few subsequent frames (here two past frames are used).

When the boundaries of the objects become slightly straighter, small gaps in the outline can be closed. While temporal integration (λ = 0.3) can improve the shape of motion discontinuities and also slightly weaken temporary outliers, it cannot eliminate them. For this reason, motion discontinuities as shown in Fig. 12D still contain some wrong estimates, in particular in the upper left and the lower right part of the image. The important role of the occlusions for segmentation based on the motion discontinuities is indicated by the results shown in Fig. 12E. Here, the segmentation after the interaction between motion discontinuity detection and occlusion detection is shown. As in experiment 5, erroneous estimates can successfully be eliminated. Thus, the interaction between the two mechanisms leads to a correct segmentation of the scene.

## Discussion

We presented a biologically inspired model for motion estimation, the detection of motion discontinuities as well as the detection of occlusion regions. This work is based on a former model proposed by Bayerl & Neumann [14] for motion detection and integration of spatio-temporal changes and object movements. Aiming at an explicit segmentation and first interpretation of the scene we extended the model by incorporating new mechanisms of spatial and temporal contrast detection of local optic flow.

### New contributions

We propose a model for the detection of both motion discontinuities and occlusion regions using different mechanisms at distinct processing stages. The whole architecture is biologically inspired, and provides a common processing principle within all model components, namely a three level processing cascade (Eq. 1–3). The modulatory feedback connections, that exist between different model components and allow the transfer of information via a “soft gating” mechanism, are crucial for the functionality of the model. This mechanism is used to stabilize the occlusion and the motion discontinuity regions. We suggest that mutual interaction between their representations makes the detected regions more reliable. Furthermore, we show that the idea of temporal integration for these regions is–again both for the occlusion and the motion discontinuity detection - a mechanism to get more robust results. Form information is used as an additional cue to improve the results. Nevertheless, as they are used as modulatory input, also stimuli without luminance contours can be processed successfully. By evaluating the motion discontinuities and occlusion regions, we derive ordinal depth order and get a coarse classification of the objects detected in the scene, whether they are static or moving independently within their local environment.

### Related work

There exist several other approaches for the detection of occlusions and for segmentation based on optic flow estimates. Ogale et al. proposed a geometric approach [2] for motion segmentation using occlusion regions. According to their method, optic flow estimates need to be computed for image pair t_−1_ and t_0_ in both forward and backward direction (t_0_/t_−1_ and t_−1_/t_0_). Regions without motion estimates are classified as occlusion regions. The occlusion regions are then filled using the already segmented results of the last and the next time step for occlusion and disocclusion regions, respectively. In an iterative processing, the segmentation of the optic flow is achieved by computing the “motion valleys” with a 3D motion estimation technique. First of all, by chosing the flow vectors of a subset of the image positions the mean background flow is estimated. At positions where the local flow is different from this mean flow, objects are segmented. Ordinal depth information is computed in a similar way as we have explained in the Methods section. We are basically following the general idea they use. The region that contains the occlusion is in the background, the adjacent region is the occluding object. Using the depth relations, objects with occlusions that were not segmented when comparing optic flow (because their flow is similar to the background flow) can now be detected. However, the approach is not able to detect objects without occlusion regions (i.e., they are in front) that are moving in similar directions like the objects behind without additional information, e.g., via a disparity estimation. In contrast, our approach for segmentation is relying on other cues. Motion discontinuities and not the flow estimates itself are used to find the regions that belong together. In other words, we suggest a boundary oriented mechanism while Ogale et al. propose to utilize the results of prototyped region segmentation to derive the ordinal depth order and object segmentation.

Recently, Ogale & Aloimonos [26] presented a compositional approach aiming at correspondence finding for stereo and optic flow estimation that includes the detection of occlusions and correct segmentation also for complex shapes. They claim that early visual modules are mutually connected to provide a means for linking different processing mechanisms to solve, for example, the chicken-and-egg problem of motion detection and segmentation. In their geometric approach they use phase-differences of local gabor filters applied for the local image structure as a matching criteria. Flow estimation, occlusion detection, and segmentation are then obtained in an iterative process of finding the largest connected regions in the image wherein a particular shift has provided the region with very high matching values. Positions that are not included in these regions, because no match is found, are labeled as occlusion positions. The segmentation can be directly derived from the regions that have the largest connected component size. The algorithm is basically contrast-invariant as the phase-difference of gabor filters is used. Only small filters are necessary as they are not used to compute the correspondence directly, but simply as a local description measure. This allows a very high spatial resolution.

The general idea of a compositional approach is also picked up in our model, but realized in a different way. While Ogale & Aloimonos use a geometric approach to find the corresponding regions, we base our model on biologically inspired processing stages that work in parallel, but share some of their information due to modulatory connections. Unlike their approach we suggest boundary processing as key for object segmentation. Mutual interactions between motion discontinuities and occlusion/disocclusion detection based on temporal center-surround competition can be applied to stabilize boundary detection. Furthermore, in their model no explicit segmentation of moving objects is computed, but only regions that share the same or similar flow.

Niyogi [4] proposed an approach for kinetic occlusion detection that is based on spatio-temporal junction analysis. Here, a biolgocially inspired distributed representation of motion is used. The changes of direction of motion in these representations are detected using an extension of an “end-stopping” mechanism applied in 2D image junction analysis. In contrast to our model, their image segmentation approach is entirely based on occlusion detection. This means that motion boundaries cannot be detected at positions where no occlusions are produced, for the movement is parallel to the object outline. In contrast, our approach detects the whole object outline in a stable way. Furthermore, the filters applied for the spatio-temporal junction analysis need several frames from both past and future time steps, which brings about a delay in processing. We avoid a long processing delay by requiring only one future and one past frame.

Recently, Feldman & Weinshall [6] also presented a model for motion segmentation and depth ordering that is based on the detection of kinetic occlusions. The mechanism uses a spatio-temporal structure tensor. Computing the eigenvalues of this tensor, the smallest eigenvalue λ_min_ is a measure whether a junction in the XYT-space is present. Furthermore, when considering the values of λ_min_ in the local neighbourhood, the position of the local maximum relative to the object boundaries is an indicator for the local depth order. Their algorithm computes the occlusion regions and depth order based on only two frames, with further stabilization if an additional third frame is available. Like the approach of Niyogi, the segmentation of this algorithm is completely relying on occlusion regions. As mentioned before, this restricts a correct segmentation to scenes including objects where the whole outline produces occlusions. Furthermore, when relying on the smallest eigenvalue, occlusion regions can only be detected for strong 2D contrasts (as a junction both in space and time is necessary to lead to values >0 for all three eigenvalues). Along 1D contrasts, the occlusion detector will not respond and thus miss possible occlusions.

In our approach, also at positions where the aperture problem occurs, the problem of motion detection and optic flow based segmentation can be solved. The V1_Model_ and MT_Model_ Motion interaction can propagate salient movement from the 2D salient positions along edges, independently of object texture. Then, MSTl_Model_ neurons can detect the motion discontinuity between object and background.

Another problem that has to be taken into account in the approach of Feldman & Weinshall is that the value of the eigenvalues is contrast dependent. For very low contrast, the response will also be very low, so that occlusion regions at the transition of two low-contrast textures might be missed. Our model has an initial motion detection that will respond to very small luminance contrasts. The dependency to local contrast is very small, as the structure but not the contrast itself are the features that we use to find matches.

### Mechanisms for improved object segmentation

We propose new mechanisms to make the segmentation of moving objects in the presence of self-motion more reliable. For this purpose, we use the computation of two scenic properties that are obtained independently, but with both representing a moving object at this position.

First, an object that is moving in front of a background will generate occlusion regions along parts of its boundaries. For the detection of the occlusion regions we propose a detector that is based on a motion energy comparison of two succeeding frame pairs, as explained in the Methods section. This approach has the advantage that it relies on the local image structure, which makes it less sensitive to contrast changes than approaches based on the detection of junctions in the spatio-temporal activity space. However, successful detection of occlusion regions is not sufficient to determine the boundary of a moving object. No occlusion will appear along the contour of an object that is moving parallel to the orientation of its outline. For that reason, approaches for object detection that are simply using occlusion detectors will not be able to gain the full object outline. As a consequence, compensation is needed.

Second, a moving object stands out due to the transition that is generated at the motion boundary; a motion boundary appears as the object motion abuts on the background motion. We use MSTl_Model_ on-center-off-surround neurons to detect the motion discontinuities based on the flow of MT_Model_.

This kind of motion boundary estimation tends to generated false estimates. If some of the MT_Model_ Motion neurons are erroneously active, strong responses in MSTl_Model_ neurons are generated. The occlusion regions provide help to deal with this problem. As explained before, a moving object will, apart from few exceptions, always lead to an occlusion region. Hence, no occlusion region can be found that is adjacent to a detected and grouped motion discontinuity, this is a strong hint for a false detection. In our approach, we included an interaction mechanism that combines the responses of the grouped motion discontinuity with the grouped occlusion regions. The motion discontinuity will be kept only if they partly abut (compare experiment 5 and 6). At the same time, the motion discontinuities also improve the results for the occlusion regions, as shown in experiment 5. The responses get more localized and many outliers are eliminated. The two interactions between the spatial and the temporal contrast detection for the estimated optic flow can so mutually improve their results.

Besides the interactions between the detection of occlusions and of motion discontinuities, we improve the results using temporal integration for the activity represented there (see experiment 6). Such an integration can be used for the different features computed in the neural model. First, it can be applied at the level of motion estimation to achieve subpixel movement detection as proposed in [24]. Second, the response of the motion discontinuities computed in MSTl_Model_ can be temporally integrated. Third, the response of the TO_Model_ neurons can use temporal integration to stabilize their responses. Altogether, the results for the features can be improved by the integration because noise appearing in just one frame has less influence on the results. In the case of motion discontinuities, boundaries can be closed and the contour gets straighter.

Based on these improved results, ordinal depth information for the scene can successfully be derived in an automatic way. Furthermore, we apply a simple classification approach to decide on the nature of the object. Is the object moving independently, or is it a static object for which the translational movement of the observer is generating movement of the image boundaries? For example, in the context of a navigation task this knowledge is very useful. In particular, objects that have a movement strongly differing from the background will be potentially dangerous for the observer. This is either caused by their independent movement or by a static object that is very close to the observer, while the background is still far away. To get a more detailed classification, further mechanisms could be added. Global flow estimation would help to decide on the self-motion component in the sequence, and perhaps the estimation could be improved by excluding segmented objects. Furthermore, stereo input would provide depth information that allowed the inference of the expected flow for an object (assuming that its movement is only caused by self movement). This could help to determine whether an object is an independently moving object.

### Relations of the model with primate visual system

In this subsection, we explain how some of the mechanisms used in the model that are not derived by existing biological data, are nonetheless plausible possibilities for processing in the brain or are related to confirmed neural mechanisms. We dwell on the occlusion detection using motion energies, the question of border ownership and the computation of depth structure.

The role that the detection of occlusion regions might play for motion processing is not yet clear. However, there is evidence that non-matchable regions improve the estimation of depth, contour, and surface perception in stereo images, as experiments by Nakayama & Shimojo [27] demonstrate. In our approach, we use this idea also for motion detection. The occlusion regions interact with the detected motion discontinuities to achieve more robust segmentation. For the detection of motion discontinuities, neurons with on-center-off-surround receptive field characteristics are a possible explanation. These kind of neurons were found in area MSTl of primates [12], [28], an area that succeeds MT in the cortical hierarchy and is responsible for small object detection and tracking. Neurons in this area respond strongly if the motion in the center region is different compared to the motion in the surround. This leads to large activity at motion boundaries. As shown in the Result section, motion discontinuities are well detected within this model component. Currently, we only use feedforward connections between MT_Model_ and MSTl_Model_. Feedback connections could help to strengthen the motion estimates at boundaries and further improve the results.

The detection of occlusions and motion boundaries is also related to the topic of border ownership, the question to which object a boundary between two objects belongs. Qui et al. [29] investigated the underlying neural correlates in neurophysiological experiments with macaques for static input images. They found V2 neurons whose responses were stronlgy modulated by the direction of the border ownership. Models trying to explain these mechanisms were relying on local contrasts and occlusion cues derived from spatial junctions (see [30] for an overview). We suggest that for dynamic scenes with moving objects the detection of occlusions and motion discontinuities as presented in our model are mechanisms that together solve the question of border ownership. The position of the occlusions and disocclusions is a direct indication for the ownership of the object boundary, the complete outline is provided by the motion discontinuities. Furthermore, an interaction with V2 Form would be possible to include the available form information or to transfer the information from the motion to the form pathway.

A first interpretation of the scene concerning the depth structure of the input sequence is achieved combining the inputs of MSTl neurons, detected temporal occlusions, and form information. A possible area to compute this feature could be KO, a small area located next to MT. Tyler et al. showed [31] that area KO is in particular responding to stimuli including depth structure, perceived either from disparity or motion cues. Neurons in this area might be tuned to depth occlusions, depth edge structure, or depth segmentation.

### Conclusion

We presented a biologically inspired model for improved object segmentation based on optic flow. Key mechanisms are a) the robust optic flow estimation based on three frames that computes continuous optic flow also along motion boundaries. b) The detection of motion discontinuities relying on these estimates effected by spatial on-center-off-suround RFs, that respond all along the object boundaries. c) The detection of occlusion regions relying on temporal contrast neurons. d) The interaction between the two mechanisms to erase erroneous estimates for object segmentation. e) Temporal integration within the different model components to stabilize the results in particular in noisy input sequences. Using these mechanisms in a unified architecture we achieve object segmentation in both artificial and real sequences, allowing a further interpretation of the scene properties such as coarse classification of object movement and ordinal depth order.
